# Enhancing the Thermostability of Papain by Immobilizing on Deep Eutectic Solvents-Treated Chitosan With Optimal Microporous Structure and Catalytic Microenvironment

**DOI:** 10.3389/fbioe.2020.576266

**Published:** 2020-10-02

**Authors:** Kai-Peng Lin, Guo-Jian Feng, Fu-Long Pu, Xue-Dan Hou, Shi-Lin Cao

**Affiliations:** ^1^Department of Bioengineering, School of Biomedical and Pharmaceutical Sciences, Guangdong University of Technology, Guangzhou, China; ^2^School of Food Science and Engineering, Foshan University, Foshan, China

**Keywords:** deep eutectic solvents, chitosan, immobilization, thermostability, structural changes

## Abstract

Deep eutectic solvents (DESs) have attracted an increasing attention in the fields of biocatalysis and biopolymer processing. In this study, papain immobilized on choline chloride- lactic acid (ChCl-Lac) DES-treated chitosan exhibited excellent thermostability as compared to the free enzyme. The properties of native or DES-treated chitosan and immobilized enzyme were characterized by FT-IR, SEM, surface area and pore property analysis. Like the common enzyme immobilization, papain immobilized on DES-treated chitosan resulted in a lower catalytic efficiency and a higher thermostability than the free enzyme due to the restricted diffusion. The results also revealed that DES could control the active group content, thus achieving the appropriate microporous structure of immobilized enzyme. Meanwhile, it could also help to construct the optimal microenvironment by hydrogen-bonding interaction between enzyme, chitosan, and residual DES, which are benefit for maintaining an active conformation and subsequently a high thermostability of papain. Moreover, it was found that trace DES (10 mM) significantly promoted the activity of free papain (145%). Deactivation thermodynamics study showed that the DES could enhance the thermostability of papain especially at high temperature (half-life of 7.4 vs. 3.5 h) because of the increased Gibbs free energy of denaturation. Secondary structure analysis by circular dichroism spectroscopy (CD) agreed well with the activity and thermostability data, further confirming the formation of rigid conformation induced by a specific amount of DES. This work provides a new way of enzyme immobilization synergistically intensified by solvents and supporting materials to achieve better microporous structure and catalytic microenvironment.

## Introduction

Deep eutectic solvents (DESs) are a type of fluids consisting of two or more safe and cheap components, namely hydrogen bond acceptors (e.g., halides salts of quaternary ammonium cations) and hydrogen bond donors, such as organic acids, alcohols, and so on. The hydrogen bond formation between these components is responsible for the lower melting point of the mixtures compared to the that of the each individual components (Abbott et al., 2004). Thus, DESs have attracted extensive attention as powerful solvents used in chemicals synthesis, biocatalysis, biomass processing and electrochemistry, etc., due to their low cost, biodegradability, non-toxicity and tenability (Zhang et al., 2012; Vigier et al., 2015).

Being similar to ionic liquids, DESs are effective solvents toward biomass because of their excellent dissolving ability. Various DESs were reported to be capable of dissolving biomass, such as starch, cellulose, xylan, and chitosan, and making them more available to application (Sharma et al., 2013; Loow et al., 2017; Prasad and Sharma, 2019). They could disturb the major linkages between sugars and lignin of biomass, and extract them from the raw materials (Feng et al., 2019; Tan et al., 2020). However, issues about DESs recovery and DESs residues in the biomass still exist (Ninomiya et al., 2015). More specifically, a certain amount of solvent often remains in the biomass after the treatment, which may reduce the quality of biomass and cause solvent loss. Therefore, a tedious and cost-intensive washing step is required to make the treated biomass free of DES and to obtain a higher recovery yield of the solvent (Ninomiya et al., 2015; Hou et al., 2017a). Nonetheless, trace of DES may still remain in the recovered- or regenerated- biomass after washings. Some researchers focused on the influence of the solvents residue on the DES mediated biomass treatment processes, especially for the biomass component or the activity of enzymes (Lehmann et al., 2014; Zhao et al., 2018). However, only limited attention has been paid to the effect of the DESs pretreatment of the supporting materials on the performance of the immobilized enzyme or the residual DES on the catalytic property and structure of enzyme.

Chitosan could be chemically or enzymatically derived from chitin, which is the second most abundant biopolymer in nature. Chitosan possesses excellent properties, such as biocompatibility, biodegradability, non-toxicity, etc. (Vicente et al., 2020). Hence, it has been considered to be a promising resource for developing biomedical materials and immobilized enzymes (Qin et al., 2010; Cao et al., 2015). Considering the excellent biocompatibility of DESs and chitosan toward enzymes, herein, the DESs treated chitosan was employed as the carrier to immobilize papain, the structural, morphologic and enzymatic properties of the immobilized enzyme and the related mechanism were studied, for better understanding the influence of the microenvironment of support matrix on the catalytic performance of immobilized enzyme.

## Materials and Methods

### Materials

Papain (Papain, 2.9 units/mg) from Papaya latex, was purchased from Sigma-Aldrin (China); High purity papain buffered aqueous suspension, from papaya latex, was also purchased from Sigma-Aldrin (China) (2 times Crystallized, ≥16 units/mg protein), and it was purified by dialysis and checked by SDS-PAGE before being used for CD structure analysis. Choline chloride (Choline chloride, 98%), lactic acid (97%), Nα-Benzoyl-L-arginine ethyl ester (BAEE), Nα-Benzoyl-L-argininie (BA) were purchased from Shanghai Aladdin Biochemical Technology Co., Ltd. (Shanghai, China). All other chemicals and reagents were of the highest purity commercially available and were used without further purification.

### DES Preparation

The DES, choline chloride- lactic acid (ChCl-Lac), used in this study was prepared as reported previously (Hou et al., 2017b). Briefly, choline chloride and lactic acid were mixed in a glass vial in molar ratio 1:2 and heated up to 90°C for 0.5 h with stirring until a clear liquid was obtained. Afterward, the DES was dried at 55°C for 48 h before use.

### Papain Immobilization

Papain immobilization on chitosan was conducted according to the literature with slight modification (Silva et al., 2015). One gram chitosan (untreated or pretreated with DES at 80°C for 3 h, collected without washing or with three times washing) was added into 100 mL of 1% (w/v^–1^) papain solution in phosphate buffer (0.1 mol/L^–1^, pH 7.0), the mixture was stirred (80 rpm) at 25°C for 12 h. The cross-linking process of chitosan was initialed by adding glutaraldehyde (2% v/v) at 30°C and the mixture was agitated at 150 rpm for 12 h. Subsequently, the immobilized papain were filtered and washed three times with distilled water and packed in 5°C before use. The protein content in solution was determined by the method of Bradford (1976).

### Enzyme Activity Determination

The activity of the free or immobilized papain was determined as described in the literature with slight modification (Cao et al., 2015). 2.8 mL of phosphate buffer (20 mM, pH 7.0) which containing substrate BAEE (final concentration 1 mM) and DES with specific concentrations for 5 min was preheated at set temperature. The reaction was initialed by adding 0.2 mL of papain solution (4 mg/mL) or immobilized papain together with 0.2 mL buffer. The concentration of product BA released was detected over 10 min at 254 nm against a blank sample without enzyme. One unit of enzyme activity was defined as the amount of the released BA (μmol) with 1.0 mg of enzyme per min.

### Characterizations

The morphology of samples were analyzed by scanning electron microscope (SEM, Hitachi SU8220, Japan), and the chemical structures of the samples were confirmed by Fourier transform infrared spectroscopy (FT-IR, Themor- Fisher-iS 50R, United States). Pore volume, porous size and surface area were determined by Nitrogen sorption measurement, which were performed on a Micromeritics instrument with 3Flex Version 5.00. Degassing of samples was done under vacuum at 60°C for at least 8 h prior to measurement. The surface area was calculated according to BET method. The C, H, N, and O content of the original chitosan and DES-treated one were measured by elemental analysis equipment (Elemantar: Vario EL cube, Germany).

### pH and Operational Stability of Free and Immobilized Papain

To determine the pH stability of the free and immobilized papain based on the DES-treated chitosan with three times washing was incubated in buffer solutions at pH 3, 4, 5, 6, 7, 8, 9, 10, and 11 at 30°C for 1 h separately. Then, the activity of papain was measured and the residual activity was calculated as a percentage of its initial activity. With respect to the operational stability, the immobilized enzyme was collected after each batch at 30°C and pH 8.0, washed with distilled water, and then used in the next batch. The operational stability of the immobilized enzyme was evaluated by measuring the enzyme activity in each batch.

### Thermodynamics Study of Papain

Briefly, papain solution with or without the presence of 10 mM ChCl-Lac was incubated at different temperatures, the samples of supernatant were withdrawn (200 μL) at intervals and immediately subjected to the activity analysis to obtain the residual activity. According to two-stage theory and first-order kinetic model, thermodynamic parameters, such as first- order deactivation rate constant (k_D_) and half-life time (t_1__/__2_) was obtained by non-linear fit using Sigma-plot 12.0 software based on the data of the residual activity of enzyme vs. time (equation: lnAr = k_D_t; Ar is the residual activity of enzyme, k_D_ is first-order deactivation rate constant and t is the deactivation time of the enzyme. From the plot of lnAr vs. t, the slope gives the value of k_D_). Gibbs free energy of denaturation (△G) was calculated by the equation: △G = −RT ln{(k_D_h)/(k_B_T); where T, h, k_B_, R were temperature, Plank’s constant, Boltzman’s constant and universal gas constant (Xue et al., 2017). The assays were repeated at least in duplicate.

### CD Spectroscopy

The secondary structures of the enzyme were analyzed on a Circular dichroism (CD) spectrometer (Chirascan, Applied Photophysics Ltd., United Kingdom) in the regions of 195–260 nm at 25°C. According to statistical methods implemented in CD software, changes in the secondary structure of the purified papain were determined in 20 mM phosphate buffer (pH = 7.0) and in the various concentrations of DES at 25°C.

## Results and Analysis

### Effect of Immobilization on the Catalytic Performance of Papain

Chitosan is available in different forms (powder, gel, fibers, and membranes), and it can be modified easily and possesses high protein affinity. Chitosan treatment could change its properties, improve its solubility in some solvents and make it more easy to process (Silva et al., 2015). Therefore, chitosan was chosen to immobilize papain by cross-linking, and the effect of DES pretreatment on the catalytic performance of immobilized papain was studied. The kinetic characters and thermal stability of the free and immobilized papain were shown in Table 1. Obviously, papain immobilized on the untreated chitosan caused the decrease of activity (86.4%) and increase of K_m_ (1.47 vs. 1.35). Also, apparently lowered catalytic efficiency was observed according to the decreased K_cat_ and K_c__a__t_/K_m_ values as compared to the free papain. An increase in K_m_ and a decrease in K_cat_ after immobilization are common in the literatures (Homaei and Saberi, 2015; Rouzbehan et al., 2017). This could be explained as the chitosan matrice may cause the steric effect of active site and limited diffusion, resulting in the reduction of enzyme flexibility which is necessary for substrate binding (Homaei, 2015; Shojaei et al., 2017). Moreover, the partial denaturing of enzyme caused by the covalent bonding reaction during the immobilization process may be also responsible for the lowered catalytic efficiency. Nevertheless, the deactivation rate constant (k_D_) decreased for the immobilized enzyme. It means that chitosan could provide a frame to keep the rigid conformation of the protein from severe distortion, preventing the unfolding of protein and deactivation of the enzyme and prolonging its half-life. These were in lines with others reports (Ahmed et al., 2020).

**TABLE 1:** The catalytic properties of free or immobilized papain.

	**DES residue^a^ (mM)**	**Relative activity (%)**	**K_m_ (mM)**	**V_max_ (mM/min)**	**K_cat_ (s**^–^**^1^)**	**K_cat_/K_m_ (s**^–^**^1^ mM**^–^**^1^)**	**k_D_ (10**^–^**^2^/h**^–^**^1^)**
Free papain	0	100	1.35	0.95	7.58	5.62	4.27
Immobilized papain 1	0	86.4	1.47	0.79	5.60	3.81	3.85
Immobilized papain 2	116.7	36.8	1.94	0.31	1.08	0.56	4.12
Immobilized papain 3	10.5	97.3	1.38	0.92	5.69	4.12	2.48

**^*a*^The amount of DES in the immobilization solution after chitosan complete dispersion and dissolution. Immobilized papain 1: Chitosan without pretreatment with DES. Immobilized papain 2: Chitosan pretreated with DES and without washing. Immobilized papain 3: Chitosan pretreated with DES and washed 3 times. The activity and Kinetic parameters of the enzymes were obtained at 30°C. K_*cat*_ is the turnover number describing the maximal substrate turnovers per second per enzyme molecule. K_*cat*_/K_*m*_ expresses the efficiency of an enzyme to bind and convert a substrate molecule.**

When chitosan was treated by DES (ChCl-Lac) before use, the catalytic performance of the immobilized papain varied with the chitosan washing conditions after DES treatment. For instance, Papain immobilization based on DES-treated chitosan without washing resulted in a noticeable decrease of enzyme activity and poor catalytic performance (lowest activity and catalytic efficiency, Table 1, 36.8% of relative activity, 1.08 s^–1^ of K_cat_ and 0.56 s^–1^ mM^–1^ of K_cat_/K_m_). The possible reason is that the high DES concentration in the immobilization solution caused distinct deactivation of papain. Interestingly, when the amount of DES residue in chitosan was reduced by washing three times, the immobilized papain exhibited significant enhancement in thermal stability (lowered k_D_ values: 2.48 vs. 3.85 and 4.12) and catalytic efficiency (larger k_cat_ and k_cat_/k_m_ values) as compared to the untreated one and unwashed one, even though its catalytic efficiency was still lower than that of the free enzyme (k_cat_: 5.69 vs. 7.58; k_cat_/k_m_: 4.12 vs. 5.62). It is likely that the DES treated chitosan or/and residual DES may exert a positive effect on papain. Understanding the underlying mechanism will be necessary.

### The Chemical Structures and Morphology of Chitosan and Immobilized Papain

**FIGURE 1:**
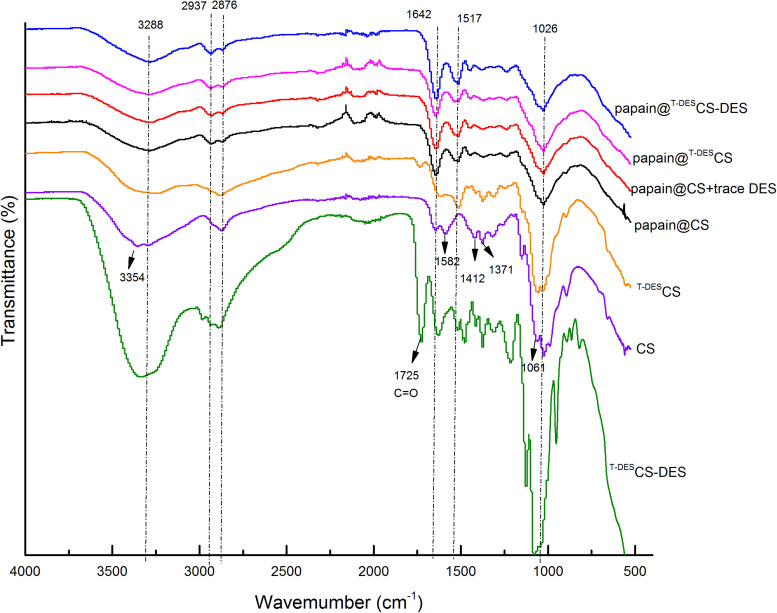
The FT-IR spectra of chitosan and immobilized papain. CS, original chitosan; ^T–DES^CS, DES treated chitosan; ^T–DES^CS-DES, DES treated chitosan with 1time washing; papain@CS, papain immobilization based on original chitosan; papain@CS + trace DES, papain immobilization based on original chitosan and immobilizing in the presence of 10 mM DES; papain@^T–DES^CS, papain immobilization based on DES- treated chitosan; papain@^T–DES^CS-DES, papain immobilization based on DES- treated chitosan with 1 time washing.

The FT-IR spectra of chitosan and immobilized papain were presented in Figure 1. Chitosan is characterized by the following peaks in IR spectra: the peak at 3,354 cm^–1^ represents stretching vibration of O-H and N-H, owing to free hydroxyl and amino groups on the side chains of chitosan; The peaks of 2,937 cm^–1^ and 2,876 cm^–1^ are related to C-H stretching; The band at 1,642 cm^–1^ is assigned to the primary amides, 1,582 cm^–1^ represents the deformation vibrations of –N–H and –C–NH_2_ groups in the amide-II region, and 1,412 and 1,371 cm^–1^ are related to the flat and non-planar deformation vibrations of NH-groups; The peaks in the 1,000–1,070 cm^–1^ region correspond to the stretching vibrations of the –CN– bonds (Holyavka et al., 2019). After DES treatment, the primary amides and NH-related peaks (1,582, 1,412, and 1,371 cm^–1^) of ^T–DES^CS became weaker compared to those of CS, indicating the reduction of amino groups in chitosan. The element analysis also proved the destruction and reduction of N content and thus the amino groups of CS (CS: *N_7_⋅_44_C_40_⋅_06_ H_6_⋅_95_O_44_⋅_02_*_VS._^T–DES^CS: *N_5_⋅_65_ C_35_⋅_94_ H_7_⋅_78_ O_42_⋅_16_*). Besides, the peak of 3,354 cm^–1^ shifted to 3,288 cm^–1^. This reveals that more intermolecular hydrogen bonds formed during the DES-treatment. When DES was presented in DES-treated CS (^T–DES^CS-DES), the clear peaks of carboxyl or hydroxyl groups (1725 and 3,354 cm^–1^) were observed. Similarly, the IR spectra confirmed the chemical composition of immobilized particles, indicating the formation of complexes and the presence of protein. The spectra of papain@CS + trace DES, papain@^T–DES^CS, and papain@^T–DES^CS-DES were similar with those of papain@CS, in which the characteristic peaks could be observed, including the C-O band in C6 at 1,026 cm^–1^, C-N stretching absorption at 1,371 cm^–1^, the primary amides at 1,642 cm^–1^ and -OH stretching presented at 3,288 cm^–1^. The differences can be identified are the shape and width of the peaks, reflecting the changes of the intermolecular or intramolecular interaction in the immobilized enzyme particles.

**FIGURE 2:**
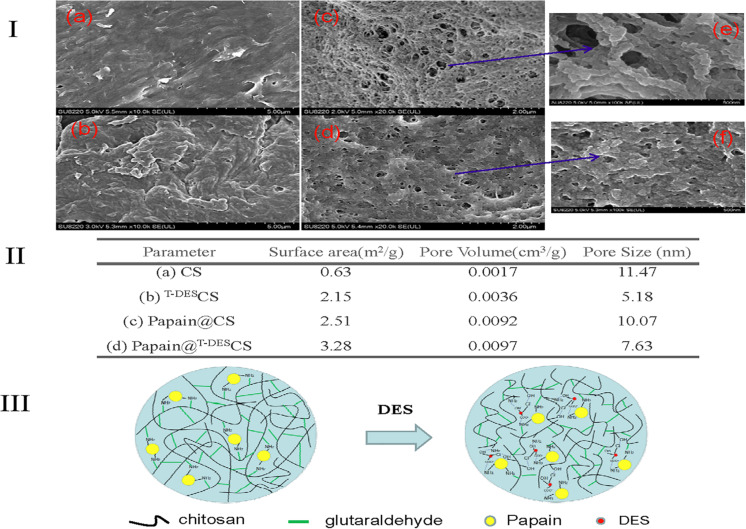
SEM micrographs of chitosan and immobilized papain **(Ia–f)**, the surface area and pore properties data **(II)** and the proposed schematic representation of enzyme immobilization **(III)**.

In addition, the morphology of the chitosan and immobilized papain were studied by SEM and showed in Figures 2Ia–f. The DES treatment resulted in more rough and hierarchical structures (Figure 2Ib) comparing to the untreated one (Figure 2Ia). It is possibly attributed to the reduction of the molecule chain of chiotsan and the stacking of small pieces. This hypothesis was further confirmed by the morphology of immobilized papain (Figures 2Ic,d). The surface of the immobilized papain based on the DES-treated chitosan became more rough than those of the native chitosan based one, and it could be clearly seen from the detailed structure in Figures 2Ie,f. Furthermore, surface area and pore size data were obtained and also shown in Figure 2II. DES treatment of chitosan (^T–DES^CS) led to the formation of larger surface area and pore volume but smaller pore size. Papain immobilization based on chitosan afforded a particle material of larger specific surface area than chitosan itself. Surprisingly, when papain was immobilized on the DES-treated chitosan, the surface area and pore volume increased, and the pore size reduced. These results are in accordance with the catalytic efficiency order of papain@^T–DES^CS and papain@CS discussed before. Therefore, the decrease of active amino group content of chitosan and the formation of appropriate microporous structure of immobilized particles caused by DES are supposed to be an important reason for the improvement in thermostability. DES participated immobilization mechanism is proposed in Figure 2III. DES may act as an adjustor to control the active group (amino group) content of chitosan in the pretreatment process and help to form the suitable degree of cross-linking during immobilization, thus achieving an appropriate microporous structure of immobilized enzyme. Meanwhile, the presence of residual DES may facilitate constructing the optimal catalytic microenvironment of immobilized enzyme by electrostatic or hydrogen-bonding interaction between DES, amino acid residues of enzyme and chitosan.

### pH and Operational Stability of Papain Immobilized on DES- Treated Chitosan

Supplementary Figure S1A shows the effect of different pH values on the stability of free and immobilized papain based on DES-treated chitosan (with three times of washing). The pH stabilities of free and immobilized enzyme were investigated in the medium with a pH value between 3 and 11 at 30°C for 1 h. The optimum pH values of free and immobilized enzyme for keeping high stability were 7.0 and 8.0, respectively. Besides, the immobilized enzyme was more stable both in acidic and basic pH ranges when compared to its free form. For example, the immobilized enzyme retained more than 40% of its activity at pH 3 and 11, respectively. Under the same conditions, only less than 20% of the activity was observed for the free enzyme. The shift in optimum pH of immobilized papain to a more alkali level has also been reported by others (Homaei, 2015; Shojaei et al., 2017). These results were mainly attributed to the diffusion limitation of the carrier (inhibiting the unfolding of the enzyme at extreme pH to some extent) and the microenvironment changes probably caused by the electrostatic or hydrogen-bonding interaction between chitosan support, residual DES and enzyme. The potential application of the immobilized papain based DES-treated chitosan was further investigated by characterizing the operational stabilities. Supplementary Figure S1B shows the activity of immobilized papain on different batches. The immobilized enzyme retained a specific activity of 82.9% after five reuses. This high operational stability indicates the promising approaches for enzyme immobilization based on the DES- treated chitosan.

### Activity of Free Papain in the Presence of DES

To further understand the microenvironment effect of DES, the effect of DES concentration on the activity of free papin was investigated. As can be seen in Supplementary Figure S2, the relative activity of papain reached to the maximum of 145.0% upon increasing the concentration of DES from 0 to 10 mM, while the relative activity still kept around 96.7% when the concentration increased to 20 mM. These results suggest that the DES may act as the activator at the concentration range of 0–20 mM, the suitable microenvironment formed with the aid of DES make the active site of enzyme molecules work efficiently, thereby increasing the enzyme activity. However, further increasing the DES concentration to 300 mM, the enzyme activity gradually decreased to 16.7%. The strong acidity of the solution formed by the presence of high concentration of DES may account for the denaturation of enzyme and thus the significant reduced activity. While others proposed that higher DES concentration could cause the destabilization of enzyme-substrate or reaction intermediate complexes according to steady-state kinetic study (Lindberg et al., 2010). In a word, within a certain concentration range, ChCl- Lac could provide an optimal microenvironment for efficiently enzymatic catalysis.

### Effect of DES on the Thermal Stability of Free Papain

**FIGURE 3:**
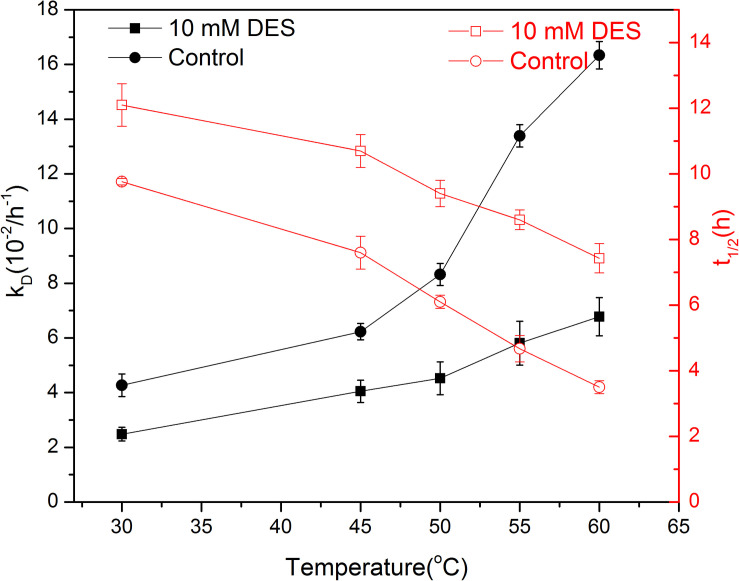
Effect of DES (10 mM) on the deactivation rate constant (k_D_) and half-life (t1/2) of papain.

In general, immobilization is an effective approach to improve the stability of enzymes (Liang et al., 2020). The k_D_ values presented in Table 1 confirmed the protective function of chitosan. Moreover, it is noticeable that there may be a synergetic effect between chitosan and DES on the thermal stability of papain. To explore detailed information about the effect of DES on the stability of papain, the thermodynamic parameters were studied. As can be seen from Figure 3, papain deactivated slowly when 10 mM DES was present, thus extending its half-life especially at higher temperature. For instance, the k_D_ value decreased from 4.27 to 2.48 when 10 mM DES was present at 30°C, and the half-life increased correspondingly from 9.7 to 12.1 h. While the k_D_ value became only 40% of the control one when DES presented at 60°C, thus achieving 2.1 times longer half-life than that of the control. These results indicate that the specific concentration of ChCl -Lac may be able to provide a suitable microenvironment by hydrogen bonding in the active center of enzyme, and then keep its rigid confirmation and function especially at a high temperature. This result is in agreement with other’s observation and conclusion that the DES could promote the thermal stability of enzymes (Kist et al., 2019). Interestingly, DESs not only can stabilize the enzyme at high temperature, but some of them also have been reported to increase the laccase stability at low temperature. The author concluded that the DES aqueous solution has a strong effect on the crystallization/freezing and melting process of water, which can reduce the number of ice crystals and hence ice crystal damage in enzyme (Toledo et al., 2019). This finding also proved that DES may be helpful for providing an ease environment together with the environmental matrix (water, immobilization materials, etc.), thus forming stable structure and function of enzyme molecule.

Gibbs free energy of denaturation (ΔG) was a reliable indicator for evaluating thermostability of enzyme, and it was proposed that the smaller the ΔG value, the lower the thermostability of the enzyme (Xue et al., 2017). The ΔG was obtained based on DES free and 10 mM DES systems. As shown in Supplementary Figure S3, ΔG values of 10 mM DES associated cases, namely 350.6, 367.3, 373.3, 379.4, and 385.6 KJ/mol, were more than those in the DES-free ones at 45, 50, 55, and 60°C, respectively. Moreover, the values apparently increased in the presence of DES at higher temperature, which is in according to trends of the k_D_ and half-life data. Based on the results, papain was more thermostable in 10 mM DES contained condition than the DES-free one.

### Effect of DES on the Secondary Structure of Papain

In order to explore the mechanism of the alteration of the thermal stability caused by DES, changes in the secondary structure of papain in the presence and absence of ChCl-Lac were investigated by circular dichroism (CD) (Supplementary Figure S4 and Supplementary Table S1). The secondary structure contents for the native papain were 24.6% α-helix, 27.4% β-sheets, and 34.4% random coils. When 10 mM DES was present, these contents were changed to 26.50, 25.0, and 32.40%, respectively. There results indicate that relatively higher α-helix content with lower β-sheets and random coil contents are favorable for the enzyme to maintain its activity, which is in accordance with the excellent activity and thermostability at 10 mM DES. Further increasing the DES concentration to 60 mM, the α-helix content reduced to 25.9%, the β-sheets slightly increased to 26.5% and the random coils almost keep constant (32.00%). The results suggest that excessive DES in the enzyme solution could denature the enzyme to a certain extent, resulting in lowered enzyme activity. All these features match very well with the activity data (Supplementary Figure S2). Similar with the finding reported by another research group (Wu et al., 2014), whose results illustrate that the specific amount of DESs may be capable of maintaining a relatively high α-helix content and thus the rigid structure for the enzyme, promoting the catalytic performance of enzyme.

## Conclusion

DES treatment of immobilization material chitosan could enhance the thermostability of immobilized papain due to formation of optimal microporous structure and catalytic microenvironment. DES could not only react with chitosan to reduce the amino group content for achieving appropriate microporous structure during immobilization, but also construct a proper microenvironment by hydrogen bonding interaction for forming rigid conformation of enzyme. The free enzyme exhibited an excellent activity (relative activity of 145.0%) and thermal stability in the presence of 10 mM DES (2 times longer for half time). Thermodynamics study shows that the presence of particular amount of DES could increase the denaturation Gibbs free energy of papain, thus making it more stable especially at a higher temperature. Results from CD analysis reveal that the specific DES concentration could promote the change of secondary structure, especially the increase of the α-helix structure content, ultimately contributing to the rigidity of the enzyme and the stability in turn. This work proves that DES not only could be used as a green solvent to improve the activity of enzymes, but also could act effectively as the processing solvent to enhance the function of immobilization material; it may synergistic work with the biopolymer materials to form a proper microenvironment with the amino acids residues, thereby promoting the thermostability of enzyme.

## Data Availability Statement

All datasets generated for this study are included in the article/Supplementary Material.

## Author Contributions

K-PL and X-DH conceived and designed the experiments and wrote the manuscript. K-PL and G-JF performed the experiments. F-LP, X-DH, and S-LC analyzed the data. All authors contributed to the article and approved the submitted version.

## Conflict of Interest

The authors declare that the research was conducted in the absence of any commercial or financial relationships that could be construed as a potential conflict of interest.
