# Supplementary material for: Enhancing the Thermostability of Papain by Immobilizing on Deep Eutectic Solvents-Treated Chitosan With Optimal Microporous Structure and Catalytic Microenvironment
Source: Front Bioeng Biotechnol. 2020 Oct 2;8:576266. doi: 10.3389/fbioe.2020.576266 (PMC7561714; doi:10.3389/fbioe.2020.576266)
Supplement: Supplementary file 1 [file Data_Sheet_1.doc]

**Supplementary materials**

**Enhancing the thermostability of papain by immobilizing on deep eutectic solvents-treated chitosan with optimal microporous structure and catalytic microenvironment**

Kai-Peng Lin^1,a^,Guo-Jian Feng^1, a^, Fu-Long Pu^1^, Xue-Dan Hou^1^, *Shi-Lin Cao^2^

^1^Department of Bioengineering, School of Biomedical and Pharmaceutical Sciences, Guangdong University of Technology, Guangzhou 510006, China.

^2^School of Food Science and Engineering, Foshan University, Foshan 528000, China.

^a^ equal contribution authors

*** Correspondence:***Xue-Dan Hou, Tex.: +86-020-39322172. Email: houxd@gdut.edu.cn.


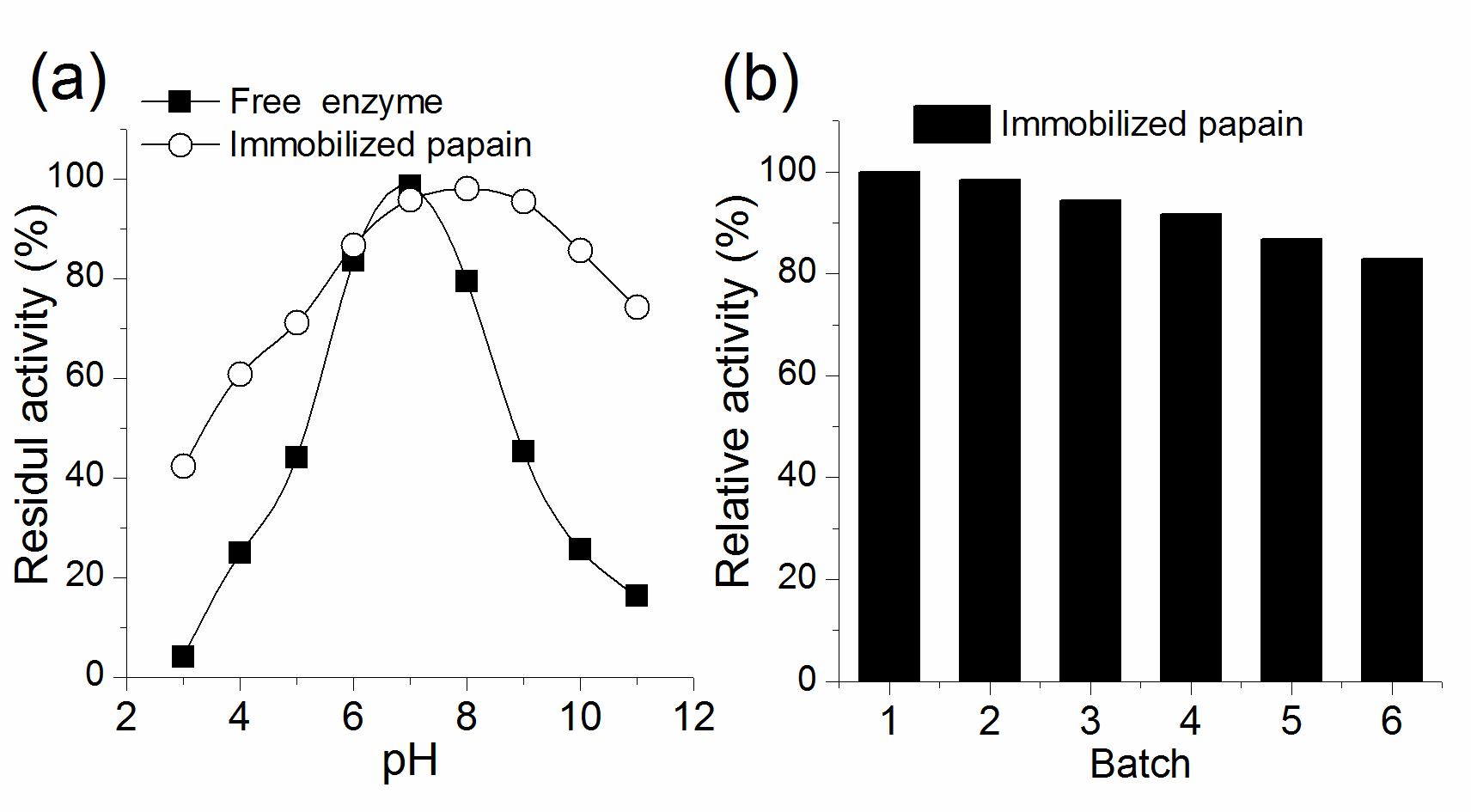


Fig. S1 pH (a) and operational stability (b) of immobilized papain based on DES-treated chitosan


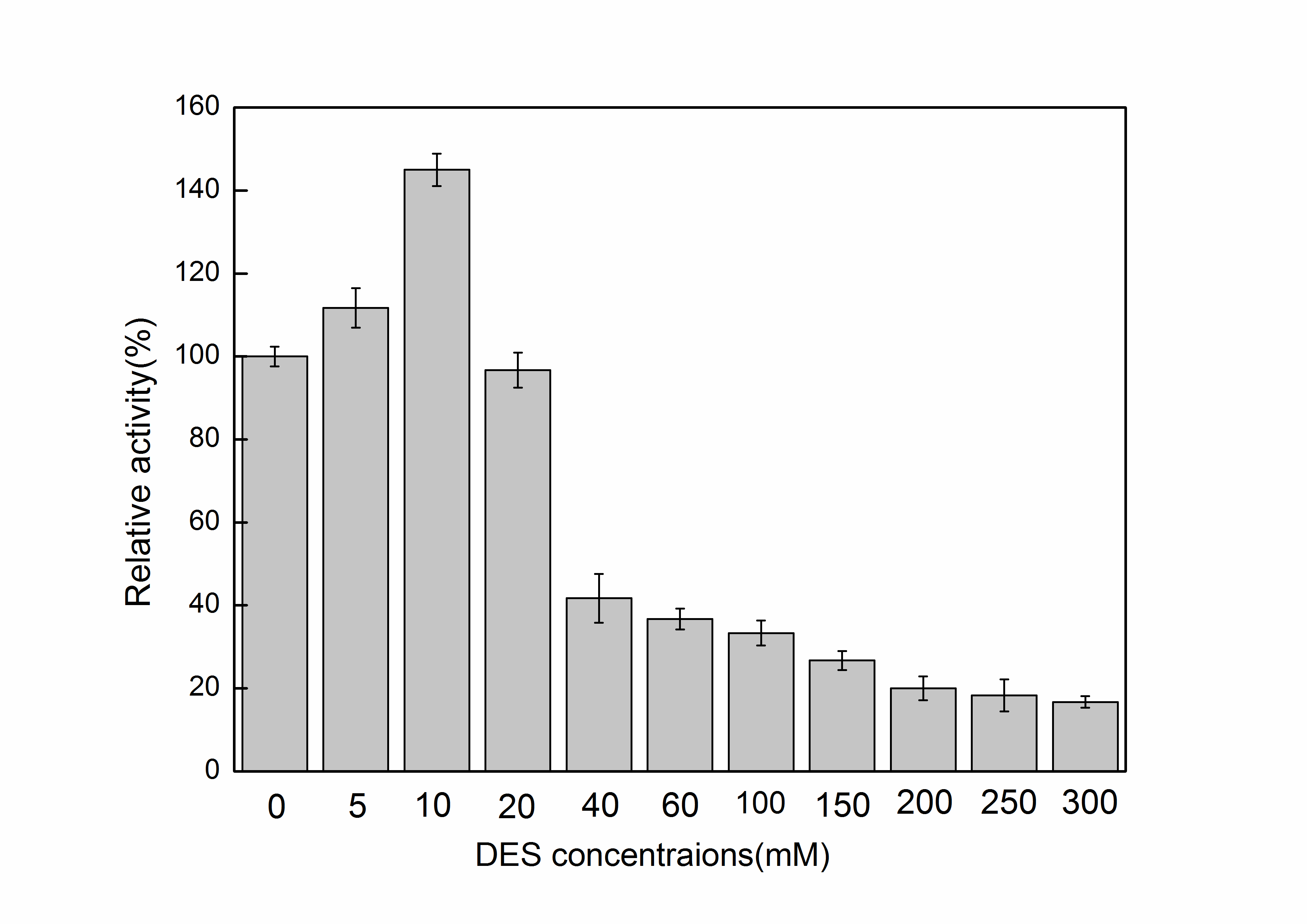


Fig.S2 Effect of DES concentration on the activity of free papain.


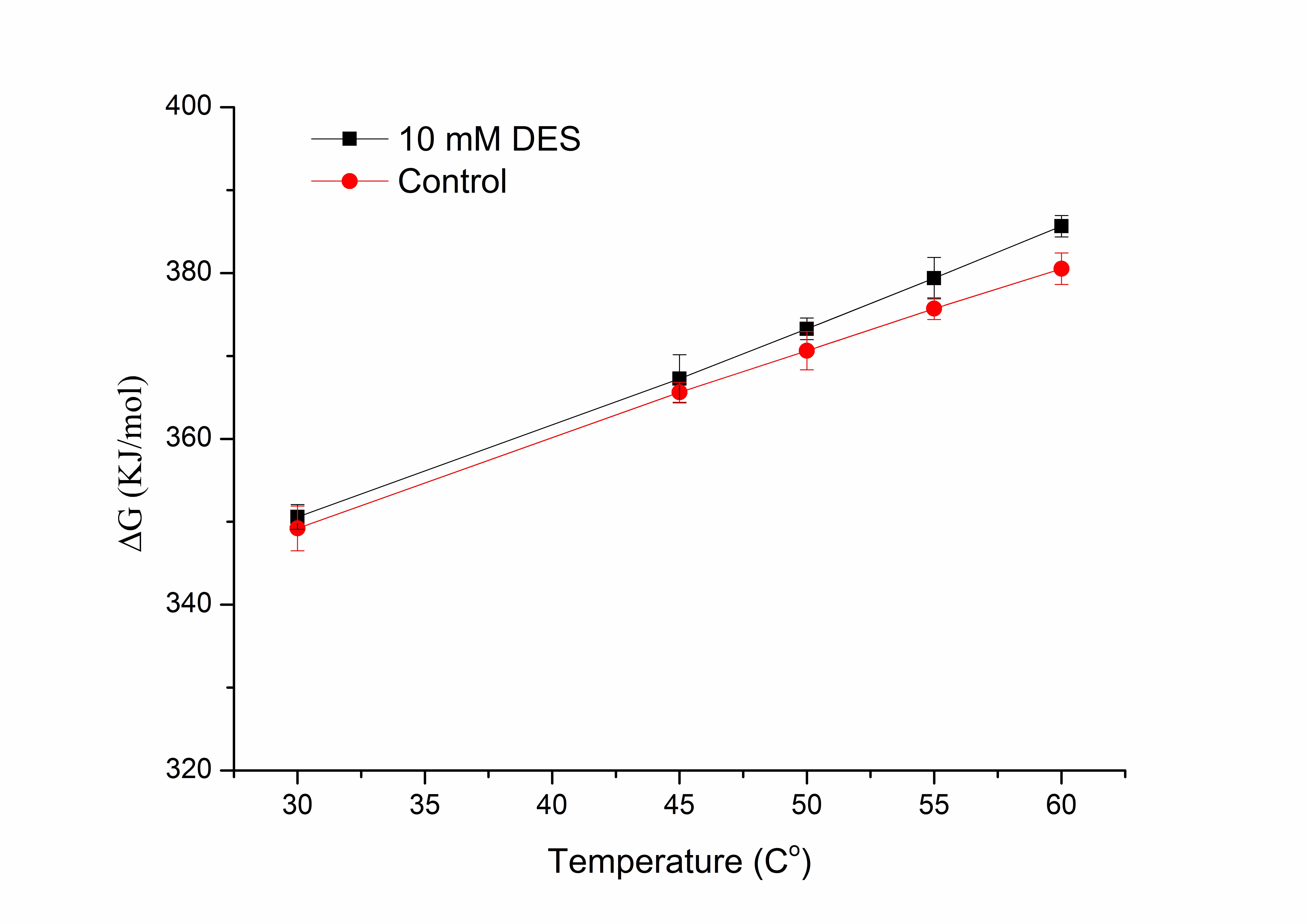


Fig.S3 Effect of DES (10 mM) on the Gibbs free energy of denaturation (△G) of papain at different temperatures


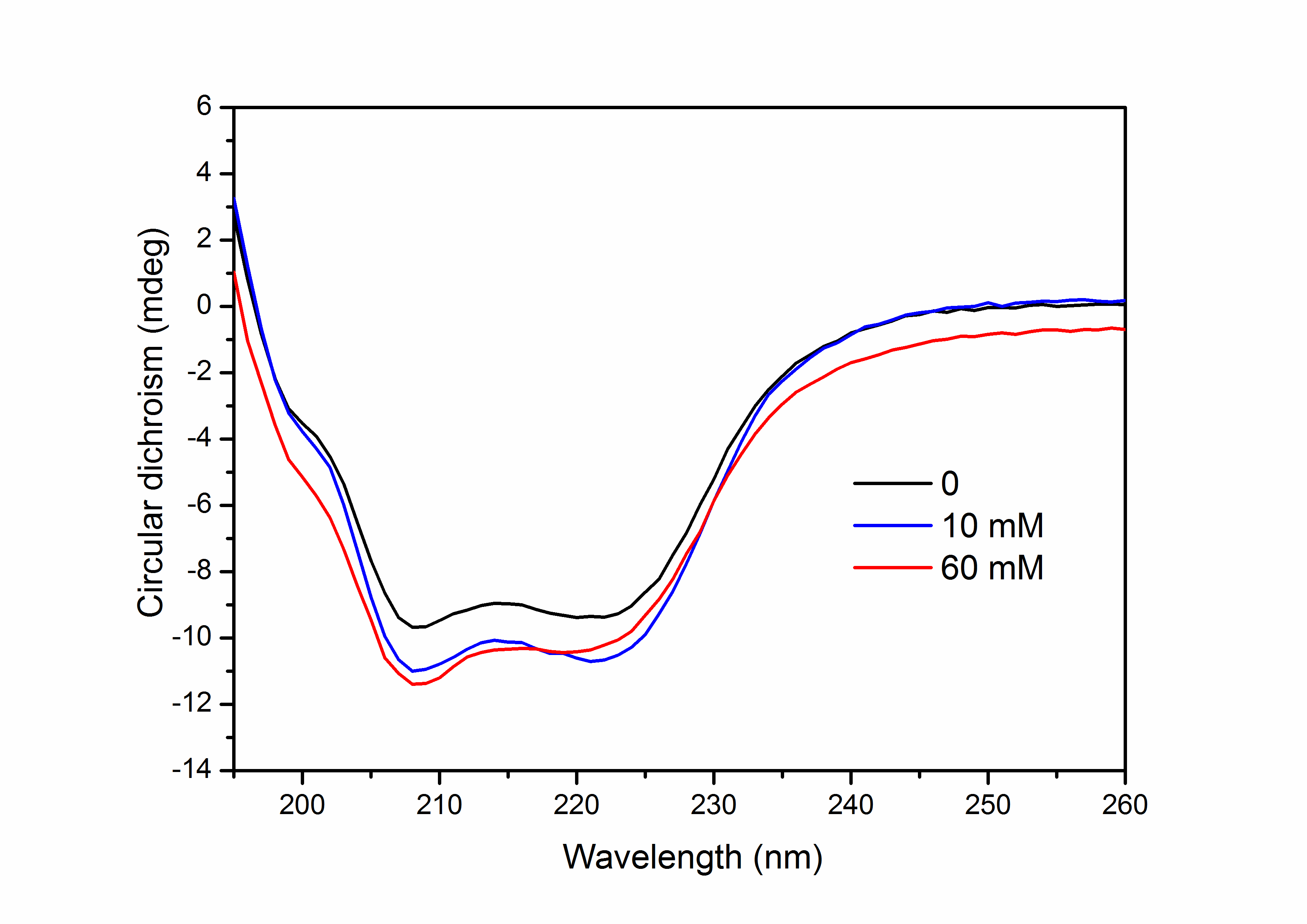


Fig. S4 CD spectra of papain in the solution containing different concentrations of DES

Table S1 The secondary structure content of papain pretreated with different concentrations of DES at 25°C

| DES concentration (mM) | α-Helix | β- sheets | Rndm. Coil |
| --- | --- | --- | --- |
| 0 | 24.60% | 25.0% | 34.40% |
| 10 | 26.50% | 27.4% | 32.40% |
| 60 | 25.90% | 26.0% | 32.00% |
